# Supplementary material for: Habits and Persistent Food Restriction in Patients with Anorexia Nervosa: A Scoping Review
Source: Behav Sci (Basel). 2023 Oct 25;13(11):883. doi: 10.3390/bs13110883 (PMC10669471; doi:10.3390/bs13110883)
Supplement: Supplementary file 1 [file behavsci-13-00883-s001.zip › behavsci-2623570-supplementary/Supplementary S2.pdf]

Supplementary S2: Strategy for the search in databases (Literature search: 19 August 2022)

"Anorexia Nervosa"[Mesh] OR "Anorexia Nervosa" OR "Anorexia Nervosas" OR "Atypical Anorexia nervosa") AND ("Habits"[Mesh] OR Habit* OR "Habit Disturbances" OR "Habit Disturbance" OR "Learned habit" OR "Conditioned habit" OR "Habitual behavior" OR "Habit systems" OR "Habit strength" OR "Habit formation" OR "eating habits" OR "Food restriction" OR "Food restriction" OR "Food refusal" OR "Dietary restraint" OR "Dieting"
