# Supplementary material for: Habits and Persistent Food Restriction in Patients with Anorexia Nervosa: A Scoping Review
Source: Behav Sci (Basel). 2023 Oct 25;13(11):883. doi: 10.3390/bs13110883 (PMC10669471; doi:10.3390/bs13110883)
Supplement: Supplementary file 1 [file behavsci-13-00883-s001.zip › behavsci-2623570-supplementary/Supplementary S3.pdf]

Supplementary S3: Main characteristics and findings of the studies included in this review.

Table S2. Main characteristics and findings of included experimental and observational studies (n=20)

**Experimental studies**							
**Author, year, country**<br><br>**• Study design**	**Sources of evidence**	**Profile of study sample/groups**	**Age (mean±DP/range) Sex**	**Place of recruitment**	**Diagnostic criteria**	**Severity symptomatology** <u>**Restriction analysis method**</u>	**Habit analysis method**
Muratore et al., 2021, [28]USA<br><br>• Randomized clinical trial	Article	**AN with treatment**<br>*20 sessions - right DLPFC*<br>HF-rTMS Group (6)<br>**AN without treatment**<br>Sham-rTMS Group (4)	30.7±7.4 y<br>Female	NYSPI/EDU	EDA-5 for DSM-5 clinical interview	*EDE-Q*<br>*BMI*<br>Food Choice Task	Structural MRI – right DLPFC<br>Identification of the DLPFC target was achieved using the FMRIB Software Library
Favier et al., 2020, [32] France<br>(El Mestikawy and Favier 2021)[50]<br>• Experimental human models (sex, age and education level matched)	Article<br>Conference abstract	**Human**<br>AN-R (21)<br>ED-BP (10)<br>HC (25)<br><br>**Mouse**<br>Groups: control; VAChTcKO mice and VGLUT3cKO mice	AN-R: 27.8±2.0 y<br>Male (2) and female (19)<br>ED-BP: 31±2.9 y<br>Female<br>HC: 29.2±1.6<br>Male (6) and female (19)<br><br>2- to 6-month-old	Care center specialized in eating disorders (inpatients and outpatients)<br><br>Phenomin - Institut Clinique de la Souris (iCS)	AN-R and ED-BP: DSM-5 criteria<br>HC: MINI<br><br>Activity-based anorexia (ABA) model	*EAT-40*<br>*EDI-2*<br>*EDQ*<br>BMI<br><br>Food intake<br>Weight	The slip-of-action neurocognitive test<br><br>Touchscreen behavioural tasks and operant sucrose self-administration

and animals							
Dalton et al., 2020, [33]UK<br><br>•<br>Randomized clinical trial	Article	**Baseline**<br>AN-GD (34)<br>HC (30)<br><br>**Treatment for AN-GD**<br>*20 sessions on left DLPFC*<br>Real rTMS (13)<br>Sham-rTMS (13)	AN-GD: 27 (11.5) y<br>HC: 25 (5) y<br>Female<br><br>30.2 ± 10.0y<br>31.3 ± 12.5y<br>Female	AN: Specialist eating disorder services and via online advertisements<br>HC: online and poster advertisements at King's College London	DSM-5 criteria<br>*EDDS*	*EDE-Q*<br>*BMI*<br>Food Choice task	Structural MRI scan – left DLPFC
Steinglass et al., 2018, [39]USA<br><br>•<br>Randomized clinical trial	Article	**REaCH +** Inpatient Behavioural Treatment (11)<br><br>**Supportive Psychotherapy +** Inpatient Behavioural Treatment (11)	30.4 ± 10.8y<br>Female<br><br>33.6 ± 10.0y<br>Female	NYSPI/EDU	DSM-5 criteria<br>EDA-5	BMI<br>*EDE-Q (global symptoms and dominions*)<br>MIM (Multi-item laboratory meals)<br>MOS (Mealtime Observation Scale)	SRHI – total strength of habit and by domains<br>SRBAI - Self-Report Behavioural Automaticity Index
**Observational studies**							
**Author, year, country**<br><br>**•Study design**	**Sources of evidence**	**Study sample (n)**	**Age (mean±DP/range) If**x	**Place of recruitment**	**Diagnostic criteria**	**Severity symptomatology**<br>**Restriction analysis method**	**Habit analysis method**
Murray et al., 2023, [26]<br>USA	Article	AN- weight-restored (29)<br>HC group (27)	AN wr: 14.8±1.7y<br>HC:16.4±1.8y<br>Female	AN: Eating Disorders Unit at UCLA and	DSM-5 criteria<br>EDE global and restraint	*YBC-EDS (compulsive subscale)*	White matter neuritis orientation and dispersion density

• Transverse				residential/partial hospital treatment centers<br>HC: Advertisements	MINI		imaging (NODDI) and analytical model of absolute tissue density from NODDI (ABTIN).<br>ROI: supplementary motor area - putamen
Seidel et al., 2022, [27]Germany<br><br>•Paired transverse (age)	Article	Acute AN (57)<br>HC (57)	AAN: 15.89 ± 2.05 y<br>HC: 17.46 ± 3.01y<br>Female	AN: Eating disorder programs of a German university/ psychiatry and psychosomatic medicine department<br>HC: advertisement among middle school, high school, and university students	DSM-5 criteria<br>SIAB-EX (modified version)	*BMI*<br>*EDI-2*	SRHI
Foerde et al., 2021, [31]USA (Foerde et al. 2020; 2022[51,52])<br><br>• Longitudinal	Symposium abstract<br>Article<br>Article	**Baseline**<br>AN (35)<br>AN 'subthreshold' (19)<br>HC (36)<br>HC-Diet (20)<br><br>**T2**<br>AN (24)<br>HC (29)	AN:26.7±6.6y<br>sAN: 23.5±5.9y<br>HC:25.8±5.3y<br>HC-D:26.0±5.4y<br>Female<br><br>AN:26.9±6.5y<br>HC:25.8±5.2y	AN: NYSPI/EDRU<br>sAN: NYSPI/outpatient<br>HC: NI	DSM-5 criteria<br>EDA-5	*BMI*<br>*EDE-Q*<br>*MIM*<br>Weight suppression and TFEQ<br>Food Choice Task<br><br>Food Choice Task	fMRI-BOLD (blood oxygenation level-dependent). ROIs: DLPFC, right anterior caudate, VMPF<br><br>fMRI-BOLD (blood oxygenation level-dependent)

Tadayonnejad et al., 2021, [30]USA (Tadayonnejad, et al. 2019; [49]Tadayonnejad et al. 2021 [30]) •Paired transverse (age)	Article Poster	**Adult study** AN-R weight-restored (20) HC (30) <br>**Adolescent study** AN-R weight-restored (12) HC group (13)	AN-R: 21.0 ±4.6 y Female HC: 22±4.7 y Male (5) and female (25) <br>AN-R :15.0±1.8 y HC: 15.0(±1.6) y Female	AN: local specialized treatment centers, online and community-based advertisements, and campus flyers. HC: NA AN: inpatient eating disorder unit at UCLA and from local treatment centers HC: NA	DSM-5 EDE	BMI YBC-EDS (severity of eating- and body/weight-related preoccupations and rituals)	Structural and difusion MRI data acquisition with voxel-based morphometry analysis and tractography Areas: Putamen, remotor/SMA: premotor/supplementary caudate motor, VMPFC
Steinglass et al., 2021, [29]USA <br>•Transverse	Conference abstract	AN (80) HC (35)	12 – 18y Female	NA	NA	Food Choice Task	fMRI. ROIs: mesolimbic reward circuits (bilateral NAcc, ventral tegmental area, and OFC) and dorsal frontostriatal habit circuits (bilateral DLPFC, dACC, and dorsal striatum).
Davis et al., 2020, [9]USA <br>• Transverse	Article	AN (69) HC (47)	AN:23.6±9.6 y HC: 23.0±5.9 y Female	NYSPI/EDU	DSM-5 criteria EDA-5	*BMI* *EDE-Q (global symptoms and dominions)* MIM (Multi-item laboratory meals)	SRHI – total strength of habit and by domains SRBAI - Self-Report Behavioural Automaticity Index

Vogel et al., 2020, [34] Germany<br><br>•Paired transverse (age and ifxo)	Article	AN (39)<br>HC (41)	AN Group: 22.7±5.1y<br>HC group: 22.2±4.1y<br>Female	AN: one of three inpatient psychotherapeutic treatment centres<br>HC: university student and general population of Bamberg, Germany	DSM-5 (structured clinical interview)	BMI<br>EDE-Q (restraint)<br>EDE-Q (total)<br>EDI – 2 (total)<br>EDI - DT (subscale "drive for thinness")	PIT paradigm
Haynos et al., 2019, [38]USA<br><br>•Paired cross-sectional (age, race/ethnicity, and education level)	Article	AN-R (19)<br>HC (19)	AN: 22.3±3.8 y<br>HC: 22.9±3.6 y<br>Female	AN: community and local eating disorder treatment facilities<br>HC: NI	SCID-IV (modified to DSM-5)	BMI,<br>Global EDE,<br>EDE restraint	fMRI in resting state.<br>ROI: bilateral NAcc, ventral caudate, and dorsal caudate seed maps
Leppanen et al., 2020, [35]USA<br><br>•Paired transverse (age)	Article	AN (46)<br>HC (56)	AN: 27.5±9.2 y<br>HC :26.3±4.5 y<br>Female	AN: the South London and Maudsley specialist eating disorders service and through online advertisements<br>HC: the local	DSM-IV<br>DSM-5<br>SCID-R version	BMI<br>EDE-Q	Anatomical MRI data acquisition (vertex-wise subcortical shape analysis and volume of the subcortical regions of interest, namely the bilateral caudate,

				community and King's College London students and staff			putamen, globus pallidus, and NAcc)
Steding et al., 2019, [37]Germany<br><br>•Paired transverse (age)	Article	Acute AN (*ac*AN) (37)<br>HC (37)	*ac*AN: 16.0±2.5 y<br>HC group: 16.2±2.6 y<br>Female	*ac*AN: eating disorder programs of a university child and adolescent psychiatry and psychosomatic medicine department<br>HC: advertisement among pupils and university students.	DSM-IV (department's semi structured interview)<br>SIAB-EX	BMI<br>EDI-2	Instrumental Motivation Task and Structural and Functional Image (fMRI). ROI: bilateral VS, mOFC, and DLPFC.
Steinglass, 2019, [36] USA<br><br>• Longitudinal paired *(age and ethnicity)*	Meeting abstract	**Baseline (T1)**<br>AN acute phase (41)<br>HC (53)<br>**After weight restoration (T2)**<br>AN (25)<br>HC (29)	AN: 27.1± 7y<br>HC:25.6±5y<br>Female	AN: NYSPI<br>HC: community	DSM-5<br>EDA-5	Food choice task	fMRI<br>Two-Step Decision Task<br>(monetary or food outcomes)
Coniglio et al., 2017, [40] USA<br><br>•Transverse	Article	AN+ atypical AN (78)	25.1±10.4 y<br>Male (2) and Female (76)	Hospital-based outpatient eating disorder clinic	DSM-5 criteria	BMI<br>EPSI: Cognitive Restraint (EPSI-CR) and Restricting (EPSI-R) subscales	SRHI of food restriction

Godier et al., 2016, [15]<br>USA and UK<br><br>•Transverse	Article	**Study 1**<br>AN (23)<br>HC (18)<br><br>**Study 2**<br>AN-R (13)<br>AN-RR (14)<br>HC (17)	AN: 25.6±6.4 y<br>HC: 31.7±7.4 y<br>Female<br><br>AN-C: 31.1±7.9 y<br>AN-R: 27,0±6,4 y<br>HC: 24.0±5.6 y<br>Female	Advertisements, the clinic website, clinician referral, and word of mouth<br><br>Via email, internet and poster advertisement, Oxford Research list for Anorexia Nervosa	DSM-5<br>EDE<br><br>DSM-5	EDE-Q<br>BMI<br><br>EDE-Q<br>YBC-EDs-SRQ<br>BMI	Slips-of-Action paradigm (Fabulous Fruit Task)<br><br>Slips-of-Action paradigm and Noise Avoidance Task
King et al., 2016, [42]<br>Germany<br><br>• Paired transverse (age)	Article	Acute AN (*ac*AN) (31)<br>HC group (31)	*ac*AN:15.7±2.5 y<br>HC: 16,1±2,4 y<br>Female	AN: eating disorder programs at a university hospital<br>HC: NI	DSM-IV (semi-structured interview)<br>SIAB-EX	BMI<br>EDI-2<br>EDI - DT (subscale "drive for thinness")	fMRI. ROI: inferior occipital gyrus; Inferior parietal lobule; L = left; CFLP = lateral prefrontal cortex; MTG = middle temporal gyrus; PCC = posterior cingulate cortex; PCL = paracentral lobule; R = right
Foerde et al, 2015, [43]<br>USA<br>(Steinglass et al., 2016; [53] Steinglass, 2016) [54]<br><br>•Transverse	Brief communications<br>Abstract<br>Symposium abstract	AN (21)<br>HC (21)	AN: 26.1±6.5 y<br>HC: 22.7±3.1 y<br>Female	AN: NYSPI/EDU (inpatients)<br>HC: advertisements	SCID<br>EDE	BMI<br>EDE-Q<br>TFEQ<br>Food Choice Task<br>MIM	fMRI (BOLD activity). ROIs: dorsal striatum (caudate and putamen), ventral striatum (nucleus accumbens), prefrontal cortex (PFC), ventro-medial prefrontal cortex (VMPFC).

							Other: amygdala, hippocampus.
Rothemund et al., 2011, [44] Germany<br><br>•Transverse	Article	AN group (12)<br>HC group (12)	AN: 24.0 ±6.1 y<br>HC: 26.0 ±3.7 y<br>Female	AN: University Hospital (inpatient)<br>HC: newspaper advertisements	NU	BMI<br>TFEQ<br>Y-BOCS	fMRI (voxel-based morphometric analysis). Brain regions: bilateral OFC, temporal gyrus, frontal gyrus, occipital gyrus, uncus, anterior lobe of the cerebellum (lingual cerebellar), precuneus, caudate body.

ABTIN: analytical model of absolute tissue density from NODDI; acAN: acute anorexia nervosa; AN: anorexia nervosa; AN-C: current restrictive AN; AN-R: recovered from restrictive AN; BN: bulimia nervosa; BMI: body mass index; BOLD: blood oxygenation level-dependent; CFLP = lateral prefrontal cortex; dACC; dorsal anterior cingulate cortex; DLPFC: dorsolateral prefrontal cortex; DSM-IV: Diagnostic and Statistical Manual of Mental Disorders-IV; DSM-5: Diagnostic and Statistical Manual of Mental Disorders-5; EAT-40: Eating Attitudes Test 40; EDA-5: Eating Disorder Assessment for DSM-5; EDE: Eating Disorder Examination; EDE-Q: Eating Disorder Examination-Questionnaire; ED-BP: binge eating/purging eating disorders; EDI-2: Eating Disorders Inventory; EDI 2 – DT Eating Disorders Inventory -subscale "drive for thinness"; EDQ: Exercise Dependence Questionnaire; EPSI: Eating Pathology Symptom Inventory; EPSI – CR: Eating Pathology Symptom Inventory - Cognitive Restraint; EPSI – R: Eating Pathology Symptom Inventory – Restricting; fMRI: functional magnetic resonance imaging; HF-rTMS: high-frequency repetitive transcranial magnetic stimulation; iCS: Institut Clinique de la Souris; L = left; MIM: Multi-item laboratory meals; MINI: Mini-International Neuropsychiatric Interview; mOFC: medial orbitofrontal cortex; MOS: Mealtime Observation Scale; MRI: magnetic resonance imaging; MTG = middle temporal gyrus; NA: not available; NAcc: nucleus accumbens; NODDI: White matter neuritis orientation and dispersion density imaging; NYSPI/EDU: New York State Psychiatric Institute; OFC: orbitofrontal cortex; PCC = posterior cingulate cortex; PCL = paracentral lobule; PIT paradigm: Pavlovian-to-instrumental transfer paradigm; R = right; REaCH: Regulating Emotions and Changing Habits; ROI: region of interest ; SCID-IV: Structured Clinical Interview for DSM-IV Axis I Disorders Screening Module; SCID-R: Structured Clinical Interview for DSM-5, Research Version; Sham-rTMS: Sham- repetitive transcranial magnetic stimulation; SIAB-EX: Structured interview of anorexia nervosa and bulimia nervosa; SRHI: Self-Report Habit Index; SRBAI: Self-Report Behavioural Automaticity Index; TFEQ: Three Factor Eating Questionnaire; UCLA: University of California; VMPFC: ventro-medial prefrontal cortex; VS: ventral striatum; YBC-EDS: Yale–Brown–Cornell Eating Disorder Scale; YBC-EDs-SRQ: Yale-Brown-Cornell Eating Disorder Scale Self-Report Questionnaire; Y-BOCS: Yale–Brown Obsessive Compulsive Scale

Table S3. Main results of included experimental, observational and reviews studies (n=35)

**Experimental studies**		
**Study, year, country**	**Goal**	**Main results**
Muratore et al., 2021 [28] USA	Test whether administering a single dose of HF-rTMS to a targeted region of the right DLPFC alters food choice behaviour in AN.	Results showed that, compared to healthy subjects, those with AN had increased activity in the right DLPFC region, and the use of HF-rTMS was associated with a reduction in fat avoidance in a food choice task ($p = 0.045$) among patients with AN, providing further support for the possibility that this region and related neural circuits may underlie restrictive food choice. There was also no significant difference in self-controlled food choices ($p = 0.12$), suggesting that this technique can be used to modulate brain activity in patients with AN.
Favier et al., 2020 [32] France<br>(El Mestikawy and Favier, 2021 [50])	Investigate the roles that acetylcholine and glutamate released by cholinergic interneurons play in habit formation and maladaptive eating.	The authors report that a subgroup of patients with restrictive anorexia nervosa was more prone to habitual behaviour than the group of healthy individuals. The response rate for the devalued outcome was positively correlated with BMI ($p=0.049$), and negatively correlated with disease duration ($p=0.77$). In addition, the exacerbated tendency of habit formation was correlated with deficit of cognitive flexibility in these patients ($p < 0.01$) and with the reduction of cholinergic activity in the dorsal striatum. In mice, genetic modification (VAChTcKO mice - no longer express VAChT), induced cholinergic dysfunction and increased maladaptive persistence ($p = 0.0002$). The inactivation of acetylcholine release by cholinergic interneurons promoted a deficit in behavioural flexibility, making them more predisposed to habit formation.
Dalton et al., 2020 [33] UK	Assess whether high-frequency repetitive transcranial magnetic stimulation (rTMS) to the left dorsolateral prefrontal cortex (DLPFC) influence food-related choice behaviour in patients with severe, enduring (SE)-AN.	At baseline, participants with severe and long-lasting AN showed a preference for low-fat foods ($p < 0.001$) and were more likely to use self-control compared to controls ($p < 0.001$). Treatment with HF-rTMS did not influence fat-related food choices, however, patients with severe and long-lasting AN had a significant improvement in food self-regulation compared to controls, in addition there was an increase in the selection of tasty and unhealthy foods ($p = 0.009$), which may be related, according to the researchers, to the effect of the treatment in interrupting maladaptive habitual behaviours.

Study, year, country	Goal	Main results
Steinglass et al., 2018 [39] USA	Test the habit model of AN by examining the impact of an intervention focused on antecedent cues for eating disorder routines.	In the exploratory analyses of each SRHI domain, there was a significant effect of the type of treatment on restrictive food intake (p= 0.015), compensatory behaviours (p=0.008) and delay in eating (p= 0.011). REACH changed the habit strength of maladaptive routines more than SPT, and a reduction in habit strength was accompanied by clinically significant improvements in eating disorder symptoms on the EDE-Q (p=0.022), in addition to an increase in the level of trend in caloric intake during the laboratory meal at the end of treatment.
**Observational studeis**		
**Study, year, country**	**Goal**	**Main results**
Murray et al., 2023 [26] USA	Estimate neurite density, orientation dispersion, and myelination of white matter tracts in habit and reward circuits in AN.	Contrary to the hypothesis, the authors did not find significant differences in neurite density (left F=0.45, p=0.508; right F=0.29, p=0.594), neurite orientation (left F=1.10, p=0.300; right F=3.29 , p=0.076) and myelin density (left F=0.54, p=0.466; right F=0.34, p=0.595) in the SMA-putamen tract, suggesting that AN cannot be characterized by white matter abnormalities between SMA-putamen in the habit loop, at least in the restored weight state.
Seidel et al., 2022 [27] Germany	To investigate ED-specific and ED-unspecific habitual behaviour and its potential relationships with eating disorder symptoms and severity in the daily lives of acutely underweight AN patients compared with HC.	In a purely restrictive sample, they showed a higher frequency of habitual behaviours compared to healthy controls. EMA outcomes indicate an increase in habit frequency, regardless of habit category. Regarding habit strength, no increase was found (as assessed with SRHI) in patients with AN, neither between nor within the two habit categories.

Foerde et al., 2021 [31] USA<br><br>(Foerde et al. 2020[52]; 2022 [51])	To assess whether food choice-related engagement of the dorsal striatum is specific to AN, or whether similar neural mechanisms are engaged by other forms of restrictive eating.	Food choices were strongly associated with dorsal striatum activity in individuals with AN, a pattern that was not found in the control group ($p < 0.05$). On average, the AN group chose high-fat foods less frequently than the HC group at both time points (p=0.00001) and these were classified as less healthy (p=0.048), this choice was associated with a decrease in in the activation of the caudate (p=0.037). Prior to treatment, the superior/precuneus parietal lobe was more involved in choice behaviour in AN compared to controls. After treatment, increased selection of high-fat foods in people with AN was associated with decreased neural activity in the caudate (p = 0.037). Changes over time in the anterior caudate and parietal lobe were significantly correlated in the AN group but not in the HC group. The authors emphasize the relationship between restrictive feeding in the AN and choice-related activation in the caudate.
Tadayonnejad et al., 2021 [30] USA<br><br>(Tadayonnejad et al. 2019 [49]; Tadayonnejad et al. 2021 [30])	Examine the structural characteristics of habitual and goal-directed decision-making circuits and their connecting white matter tracts in two independent data sets of adults and adolescents as an explanatory sub-study.	Adults with AN had a significantly greater volume of total bilateral premotor tracts/supplementary motor area-putamen (p = 0.046) involved in the habits circuit compared to the control group. Showing that patients with AN had greater connectivity in brain networks involved in habitual decision-making. In adults and adolescents, the total number of bilateral tracts (p = 0.034) and the total volume of bilateral tracts (p = 0.005) were positively correlated with the severity of symptoms of ritualistic/compulsive behaviours measured by the YBC-EDS in adolescents (p=0.037) and adults (p = 0.033) with AN. In addition, the influence of the habit loop on ritualistic/compulsive symptoms was moderated by worry symptom severity levels (p = 0.001). In the adult substudy, a significant positive correlation was found between the volume of the habit loop white matter tracts and the HAMA score (p = 0.02). No significant correlation was found between BMI and tract volume in the AN adult (p = 0.15) or adolescent (p = 0.16) group. The authors point out that the data provide support for the neurobiological model of AN based on habitual decision-making circuits.
Steinglass et al., 2021 [29] USA	Examine the neural mechanisms of food choice among adolescents with AN as compared with HC in the baseline timepoint from the	Adolescents with AN were more likely to: limit the selection of high-fat foods (p = 0.001), make choices influenced by health (p = 0.001); and classify healthier items as tastier (p = 0.001). In preliminary analyses of the fMRI subsample (42AN), the involvement of the dorsal striatum is less than the ventral one, although the difference is not statistically significant in this small sample (p = 0.100).

	longitudinal study.	
Davis et al., 2020 [9] USA	Test the habit model of AN by examining the impact of an intervention focused on antecedent cues for eating disorder routines.	Habit strength was significantly higher in subjects with AN than HC (p<0.001), was significantly correlated with disease duration (p =0.001) and with AN severity (p <0.001) as measured by EDE-Q. In patients with AN, it was not significantly associated with caloric intake in the laboratory meal (p=0.10), suggesting that habit formation is probably not the only restrictive eating mechanism.
Vogel et al., 2020 [34] Germany	Investigate Pavlovian learning and the impact of stimuli conditioned to low-calorie and high-calorie food rewards on instrumental responding for different food rewards.	The results indicated that patients with AN had deficits in acquiring knowledge of experimental contingencies (p=0.030), in addition, in this group the instrumental response to low-calorie foods increased (p<0.001) with increasing severity of psychopathology of the eating disorder assessed by the EDE-Q, suggesting a behaviour directed towards weight loss.
Haynos et al., 2019 [38] USA	Characterize the functional organization of the striatum in AN, and to begin to elucidate the relationship between dysfunctional RSFC and clinical presentation.	Individuals with AN-R presented, in relation to the control group, lower functional connectivity at rest between the NAcc and the superior frontal (p=0.001), temporal and posterior gyrus (p<0.001), as well as other dorsal striatum areas (p=0.001). Authors suggest that poor caudal dorsal connectivity with other brain regions (temporal cortex) may be associated with greater cognitive symptoms of eating disorders. Greater functional connectivity at rest in the dorsal caudate-putamen was associated with lower BMI in the AN group, with a significant initial confidence interval with repeated sampling (CI: -0.03, -0.56), which is consistent with the habit-centered hypotheses about maintenance of AN.
Leppanen et al., 2020 [35] USA	To explore the morphometry of basal ganglia regions including the bilateral caudate, putamen, globus pallidus, and NAcc in light of the reward centred model of AN	There were no significant differences between groups in the volume of the basal ganglia regions (left or right caudate volume, putamen, globus pallidus or Nacc). However, there were small group differences located in the shape of these regions, the AN group had less in the caudate (p=0.022) and globus pallidus (0.004). There was no significant correlation between EDE-Q or disease duration and vertex index in the AN group. The authors suggest that there are changes in the regions of the basal ganglia associated with habit formation.
Steding et al., 2019 [37] Germany	Test whether patients would adopt a goal-directed or habitual behavioural	The authors highlight a decrease in medial orbitofrontal cortex (mOFC) activation during reward anticipation in a group of patients with habit-oriented anorexia (hAN) (p=0.007), which would be in line

	strategy when solving a task and identified the underlying neural mechanisms.	with a habit-driven response.
Steinglass, 2019 [36] USA	Test whether complex and seemingly highly controlled behaviours reflect heightened or deficient model-based behaviour; examine the specificity of findings comparing results from food and monetary versions; evaluate patients before and after weight-restoration treatment.	The results suggest that model-based rather than model-free behaviour was impaired among individuals with AN compared to HC (p=0.023). Regardless of dietary or monetary outcomes, model-based learning was worse in AN (p=0.27). Basic mechanisms of habitual learning may be intact, while flexible responses to changing contingencies and the ability to integrate a model of the environment into choices are impaired.<br><br>Dorsal striatum involvement occurred during food choice more in individuals with AN than with HC (p<0.005). And weight restoration did not change food choices or neural substrates (p=0.30).
Coniglio et al., 2017 [40] USA	Test whether habit predicted restriction even after controlling for illness duration.	Illness duration, diagnosis, and cognitive restriction were not significant predictors of restriction, whereas habit strength was a significant predictor. Habit explains 27.9% of the variation in food restriction (p=0.001).
Godier et al., 2016 [15] USA and UK	To test if a generalised reliance on habits, may contribute to the development of the compulsive weight-loss behaviour in AN.	In sample 1, no correlations were found between task performance and any of the clinical measures or questionnaires. In sample 2 there were no significant correlations between performance on the habit test and any clinical measures/questionnaires. There was no difference between the three groups on any task in the ability to retain previously rewarded responses when the outcome is devalued (p=0.957).
King et al., 2016 [42] Germany	To investigate the neural substrate of intertemporal choice in a sample of predominately adolescent patients in their first episode of AN.	Activation associated with decision making was decreased in AN, especially in the lateral prefrontal and posterior parietal regions, which are implicated in executive control. And there was less activation in patients with AN in a dorsal anterior cingulate cortex region in the follow-up analysis of difficult decisions.

Foerde et al, 2015 [43] USA<br><br>(Steinglass et al., 2016 [53]; Steinglass, 2016 [54])	To examine the neurobiological mechanisms underlying persistent maladaptive food choices in anorexia nervosa.	Low-fat food choices were related to greater dorsal striatum activation ($p<0.05$) in adolescents and adults with AN, and this activity in frontostriatal circuits was correlated with their actual food intake at a meal in the next day ($p=0.04$). Functional connectivity analysis (psychophysiological interaction, PPI) corroborates this finding, confirming that connectivity was stronger for low-fat foods ($p<0.05$) in the AN group.
Rothemund et al., 2011 [44] Germany	Investigate whether the clinical feature of compulsivity in anorexia nervosa patients relates to regional brain activation.	Voxel-based morphometric analysis indicated a significant reduction in overall gray matter volume ($p=0.020$) and a significant increase in cerebrospinal fluid ($p<0.001$). When comparing the AN group with the control group during the hypercaloric condition, patients with AN activated the right caudate body and the right precuneus ($p<0.005$), indicating differences between the two groups regarding habit and learning mechanisms.
**Reviews studeis**		
**Study, year, country**	**Goal**	**Main results**
Mysliwiec, 2020 [10] New Zealand	Perform a manualized family-based treatment review (FBT) through a neuroscientific lens.	The review does not specifically address the habit in AN, but the implications of the neurobiological mechanisms underlying AN to improve the effectiveness of FBT in adolescents. The study approaches the importance of understanding the fear and anxiety experienced by individuals with AN in a family context. Predisposition to fear conditioning leads to behavioural avoidance with greater resistance to fear extinction learning. The authors mention that AN shares neurobehavioural patterns with anxiety and fear disorders, involving aberrations in reward processing and habit formation. Furthermore, patients with AN may have formed maladaptive habits regarding food and eating that are difficult to break without targeted intervention. Suggesting that the FBT approach may be useful to promote habit reversal in patients with AN.
Frank; Shott, DeGuzman, 2019 [12] USA	To review recent advances in the understanding of the neurobiology of AN.	The review discusses the possible involvement of dysfunction in the neural circuits of the reward system, which leads to a reduction in the ability to experience pleasure and satisfaction with food, leading to dysfunctional and inflexible food choices. This may contribute to a greater dependence on restrictive eating behaviours in AN, so the vicious cycle of these behaviours becomes automated and difficult to

		change habits, which makes it difficult for patients with the disease to recover. Therefore, the review suggests that AN may involve changes in neural circuits associated with habit and emotion regulation, which may have implications for the treatment of the disease.
Ruffin, Steinglass, 2019 [11] USA	Report how anxiety and habits contribute to anorexia nervosa	This review highlights that anorexia nervosa involves a wide variety of dysfunctional, repetitive, and stereotyped eating behaviours (for example, calorie counting, constant weighing and refusal of certain foods that can be influenced by emotional factors such as anxiety. Thus, people with AN may resort to repetitive behaviours to deal with their feelings of discomfort. In addition, a cycle of repetition of these behaviours can lead to the consolidation of dysfunctional habits and therefore make changing behaviour even more difficult.<br><br>The authors also report that restrictive eating is a learned behaviour described by patients as initially rewarding, so these experiences likely activate reward pathways in the brain (ie, ventral striatum, orbitofrontal cortex), which in turn support continued food restriction. As restrictive eating continues, goal-directed behaviours that were previously reinforced become more automated. Finally, the review emphasizes the importance of addressing both the emotional and behavioural aspects of AN to have an effective treatment that involves destabilizing dysfunctional eating habits and replacing them with healthier eating behaviours.
Uniacke et al., 2018 [20] USA	Describe the concepts of cognitive neuroscience and relevant data from research in AN.	The authors point out that the involvement of the dorsal frontostriatal circuits is behind the persistent and life-threatening food restriction observed in AN. As behaviour becomes less reward-dependent and more automated, structures associated with habit are involved, including the dorsal striatum and dorsolateral prefrontal cortex. In addition, the review points out that the formation of habits can be facilitated by emotional factors, such as anxiety and stress, and that the persistence of these habits can be impaired by cognitive processes such as the distorted perception of control and danger of food.
Tasneem, 2018 [13] Canada	To examine the advantages and limitations of two animal models that are currently used by researchers, the food restriction model and the activity-	The review proposes that the habit-centered model sees food restriction intake in anorexia nervosa as a learned habit, not innate and provoked by specific stimuli. Furthermore, it highlights that habit formation can be influenced by several regions of the brain, including the basal ganglia and prefrontal cortex, and suggests that an understanding of habit formation in AN may help inform the development of more

	based anorexia model.	effective treatments. effective for the disorder.
Lloyd et al., 2017 [41] UK	Consider the factors and mechanisms identified as relevant to reinforcement and abnormalities of the goal-directed system in other compulsive disorders to understand how they develop in AN	The authors suggest that the psychopathology of AN directly stimulates excessive repetition of food restriction that results in habit formation. The review highlights that individuals with AN may be physiologically more capable of starving themselves during the period necessary for the formation of behavioural habits, since increased 5-HT activity consequently increases satiety and anxiety. Thus, the authors propose a model in which the anxiety of individuals with AN play a central role in the development of compulsive and restrictive eating behaviours. Thus, anxiety can affect the functioning of the goal-directed system and lead to the development of a vicious habit cycle, in which anxiety triggers extreme dieting behaviour, which temporarily reduces anxiety and reinforces the habit. Over time, this habit becomes ingrained and difficult to break, leading to the development and maintenance of AN. The authors propose that directing studies to understand anxiety and the habit cycle may be a promising path for the treatment of AN.
Steinglass; Walsh, 2016 [14] USA	To review a cognitive neuroscience model of AN that focuses on the persistence of maladaptive behaviour	The review points out that restrictive eating in AN is learned and elicited by specific cues for the individual and that it is associated with activity in the dorsal striatum, which is a key prediction of the habit model of AN. The authors present a neurobiological model aiming to explain the persistence of AN. According to the authors, behaviours characteristic of AN, such as restrictive eating behaviours, body image distortion and intense fear of gaining weight, are maintained by a variety of biological, psychological, and social factors, which can become habitual. The model proposed by the authors suggests that anorexia nervosa involves a dysfunction in the brain regions responsible for regulating reward, inhibitory control, and emotion, which includes the medial prefrontal cortex, amygdala, ventral striatum, and hypothalamus. Thus, changes occur in the processing and response to food stimuli, which can lead to the formation of restrictive eating habits. Also, a malfunction in emotion regulation can lead to greater sensitivity to stress and anxiety, and to deal with emotions these individuals tend to restrict. And finally, the authors highlight the importance of decision-making since a dysfunction of the medial prefrontal cortex can lead to difficulties in choosing healthy eating behaviours and in inhibiting restrictive eating behaviours.

Compan et al., 2015 [17] France	To provide an overview of neural circuitry of restrictive food choice, binge eating, and the contribution of specific serotonin receptors.	This review does not specifically address the role of the habit system in anorexia nervosa but discusses how the brain implements adaptive decision-making related to eating. The authors suggest that maladaptive decision making leads the brain to implement a food restriction that can lead to death, and such action is related to the habit system that "store" stimulus-response associations based on past rewards, and mention that there is evidence that patients with AN have deficits in the reward learning system, which can lead to greater dependence on the habit to the detriment of cognitive flexibility and behavioural adaptation. The neural substrates involved in initiating the transition from transient to persistent restrictive food intake may center on ascending serotonergic inputs from the dorsal raphe nucleus to the mPFC. And excessive dependence on the habit may contribute to the persistence of food restriction in patients with anorexia nervosa.
Guarda et al., 2015 [45] USA	To review the relationship between anxiety and AN, including potential mechanisms by which anxiety, stress and fear learning contribute to the etiology and maintenance of this disorder and how estrogen may influence these associations	The review points out that habit formation likely contributes to abnormal eating and exercise behaviours in AN and that secondary alterations in reward-related brain circuits, along with disturbances in neuroendocrine hunger and satiety signalling, possibly contribute to the maintenance of anorectic behaviours in genetically vulnerable individuals. The authors argue that food restriction can become a habit over time and that this can be maintained by factors such as anxiety and stress. According to the authors, anxiety can be a trigger for food restriction, and maintenance of this behaviours can be reinforced by the temporary relief of anxiety that occurs after restriction. In addition, the authors suggest that food restriction in patients with anorexia nervosa can be learned through associations between the food environment and the emotional experience, such as the sense of control and power that can be achieved by restriction. Together, these emotional and learning factors may contribute to the maintenance of food restriction in patients with AN. However, the specific role of habit in food restriction is not addressed in depth in this review.
O'Hara, Campbell, and Schmidt, 2015 [16] UK	Examine the neurobiological and psychophysiological evidence supporting a role for altered reward processes in the development and maintenance of AN	This review does not directly address the role of habit in food restriction in patients with AN. Instead, the review proposes a reward-centered model to explain the mechanisms underlying AN, in which AN can be considered as a reward-based learned behaviours and habitual control of motivated behaviours increases susceptibility to automatic and habitual cognitive biases. towards AN-related concerns and behaviours (such as restriction). The authors propose a reward-centered model, in which food restriction can be seen

		as a way to search for alternative rewards. However, the review highlights that food restriction behaviours in patients with anorexia nervosa can become habitual, since the repeated activation of certain behaviour patterns can lead to habit formation. These habits can be reinforced by internal rewards, such as a feeling of control or reduced anxiety.
Godier; Park, 2014 [46] UK	Exploring how a transdiagnostic view of compulsivity can deepen understanding of persistent weight loss behaviour in AN.	Although the term "habit" is not directly addressed, the authors discuss the rigidity and persistence of restrictive and compulsive behaviours in anorexia nervosa, which can be interpreted as habitual and automated behaviour patterns. The review suggests that habit formation can be considered as a mechanism by which initially rewarding weight loss behaviour in AN can become compulsive over time, since the neural circuits involved in action-response and stimulus- response are similar to models of neurocircuitry involved in compulsiveness, which indicates that these constructs seem to be indexed by overlapping neurocircuitry. The authors emphasize the importance of understanding the relationship between compulsivity and food restriction in anorexia nervosa, suggesting that compulsive behaviour may be a way of trying to control food intake and maintain food restriction, which may perpetuate the vicious cycle of anorexia nervosa.
Park; Godier; Cowdrey, 2014 [47] UK	Describe recent research on the neuroscience of reward and compulsivity in AN.	The ability of individuals with AN to maintain food restriction can be explained in part by an imbalance between reward and inhibitory control aspects, and it can become a maladaptive habit. Excessive habit formation in AN is suggested as an underlying mechanism that perpetuates compulsive weight loss behaviour (such as food restriction). AN treatment can benefit from understanding dysfunctions in the reward system and habit formation.
Walsh, 2013 [18] USA	To present a formulation in which the marked persistence of anorexia nervosa can be understood as a well-established maladaptive habit.	The author proposes a model, based on evidence from research with humans and animals, in which the central element that explains the persistence of eating behaviour in AN is that it has become a habit, developed, and sustained by neural mechanisms based on principles of action- outcome and stimulus-response learning. Thus, he discusses that food restriction in AN can be understood as a habitual behaviour and brings that the repetition of restrictive behaviours can lead to the occurrence of an automated habit, difficult to modify. In addition, food restriction can be reinforced by non-food rewards, such as the feeling of control and weight loss, and finally it is understood that understanding food

		restriction in terms of habit can help in the development of treatments for AN patient.
Marsh et al., 2007 [48] USA	Focus on self-regulatory control processes and learning habits in AN and BN.	Disturbances in habit learning may contribute to the persistence of restrictive eating behaviours in AN. The authors present a model in which disturbances in the frontostriatal systems interact with an underlying vulnerability to develop repetitive and stereotyped behaviours and thus disrupt self-regulatory control, contributing to the development of AN. The authors suggest that food restriction in patients with AN can be understood as a result of the complex interaction between cognitive, emotional and behavioural factors, and emphasize the role of self-regulatory control and habit learning in the pathogenesis and maintenance of the eating disorder, and thus, emphasizes that the loss of self-regulatory control can lead to rigid and repetitive behaviours, which become habitual over time, and also discusses the importance of therapeutic interventions to improve self-regulatory control, decrease habitual dysfunctional behaviours and help patients achieve healthier and more flexible eating behaviour.
Steinglass; Walsh, 2006 [19] USA	To review knowledge about disorders in cognition and neurobiology associated with the persistence of habitual behaviours in obsessive-compulsive disorder (OCD) and consider how these can contribute to the understanding of AN.	Based on the neurobiological model of OCD, which involves a disorder of the implicit learning system, it is suggested that behaviours in AN, such as restrictive eating behaviour, may persist because patients are unable to unlearn this habit. According to the authors, a neurobiological model of AN is based on a prominent neurobiological model of OCD and thus allows testing whether AN involves a disturbance of the implicit learning system mediated by cortico-striatal-thalamo-cortical neural circuits (CSTC). This neurobiological disorder is also believed to underlie a deficit in implicit learning, so that patients with AN have difficulty learning new behaviours or unlearning old ones.

AN: Anorexia, Nervosa; BN: Bulimia Nervosa; BMI: body mass index; CI: confidence interval; CSTC: cortico-striatal-thalamo-cortical neural circuits; DLPFC: dorsolateral prefrontal cortex; ED: Eating disorder; EDE-Q: Eating Disorder Examination-Questionnaire; EMA: ecological momentary assessment; FBT: family-based treatment; fMRI: functional Magnetic Resonance Imaging; HAMA: Hamilton Anxiety Rating Scale; hAN: habit-oriented anorexia nervosa; HF-rTMS: high-frequency repetitive transcranial magnetic stimulation; mOFC: medial orbitofrontal cortex; mPFC: medial prefrontal cortex; NAcc: nucleus accumbens; OCD: Obsessive Compulsive Disorder; PPI: psychophysiological interaction; REACH: Regulating Emotions and Changing Habits; SRHI: Self-Report Habit Index; SMA-putamen: sensory-motor area to the putamen; SPT: Supportive Psychotherapy; YBC-EDS: Yale–Brown–Cornell Eating Disorder Scale; 5-HT: 5-hydroxytryptamine
